# *In situ* monitoring of polarons in a mixed conducting polymer using ultrafast transient absorption spectroelectrochemistry

**DOI:** 10.1039/d5sc04619j

**Published:** 2025-08-27

**Authors:** Caitlyn Clark, Abdul Rashid Umar, Christopher Grieco

**Affiliations:** a Department of Chemistry and Biochemistry, Auburn University Auburn Alabama 36849 USA czg0090@auburn.edu

## Abstract

Organic mixed ionic–electronic conducting polymers remain at the forefront of materials development for bioelectronic device applications. During electrochemical operation, structural dynamics and variations in electrostatic interactions in the polymer occur, which affect dual transport of the ions and electronic charge carriers. Such effects remain unclear due to a lack of *in situ* spectroscopic methods capable of capturing these dynamics, which hinders the rational design of higher-performance polymers. Herein, we present the first *in situ* transient absorption spectroelectrochemical measurement of a conducting polymer in the near-infrared, where photoexcited charge carrier dynamics are used to directly probe their nanoscale environment and trapping behavior in working electrodes. In this method, voltage is applied to charge or discharge the polymer, and the picosecond relaxation dynamics of directly photoexcited charge carriers are spectroscopically monitored to relate their location within the heterogeneous polymer nanostructure to their transport behavior. Applying this technique to working PEDOT:PSS electrodes, we investigated the impacts of voltage-induced changes in polymer chain packing and ion–carrier interactions on charge trapping. At lower voltages, carriers initially form within J-aggregated PEDOT chains that are deeply trapped due to strong electrostatic coupling to PSS^−^ counterions. At higher voltages, the PEDOT lamellae expand and charge–ion pairs enter the PEDOT-rich domains, where trapping is decreased and carriers delocalize among the more tightly stacked, H-aggregated PEDOT chains. Further, this *in situ* spectroscopic method can also be more broadly applied to study electrochemical dynamics in accumulation-mode and n-type polymer electrodes and electrochemical transistors.

## Abstract 2

Combining electrochemistry and near-infrared transient absorption spectroscopy, we demonstrate a new *in situ* measurement approach for monitoring charge carriers in conducting polymers.

## Graphical abstract

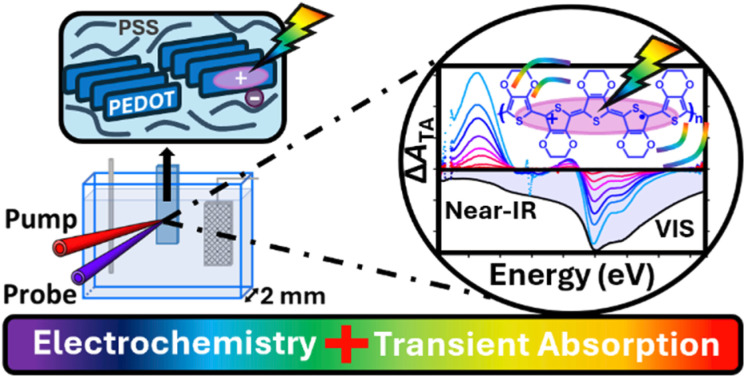

## Introduction

Organic mixed ionic–electronic conductors (OMIECs) are a unique subset of conjugated polymers that readily solvate ions and support dual transport of ionic and electronic species, making them suitable for organic electronic devices.^1–3^ Three fundamental physical processes underlie OMIEC device performance: ionic transport, electronic transport, and ionic–electronic coupling.^1^ Understanding these fundamental principles and their dependence on polymer structural dynamics that occur during device operation is important to guide the development of high-performance polymers for next-generation bioelectronic,^4^ optoelectronic,^5^ and energy storage devices.^6^ While many findings have emerged from *ex situ*^2,7^ optical, electrical, and structural studies of OMIEC polymers, in addition to myriad studies on chemically doped (dry) polymer films, this knowledge may not translate to electrochemically-active OMIEC systems.^8,9^ For example, although the electrostatic interactions of ion–hole pairs are known to significantly impact hole trapping in chemically doped polymers,^10,11^ their role remains unclear in working OMIEC devices, in which the polymer dynamically swells with ions and electrolyte. While the development of *in situ*^12–15^ and *operando*^7,16^ structural measurements continues to grow, spectroscopic measurements capable of probing structure–function relationships of the ion–carrier pairs in working OMIECs remain scarce.

Previously, Schwartz's^17,18^ and our^19–21^ groups have demonstrated ultrafast transient absorption (TA) spectroscopy as an effective optical method for probing the molecular environment and local mobility of the ion–charge pairs in chemically doped polymers. In this method, near-infrared (near-IR) photoexcitation of the charge carrier transiently separates the ion–charge pair,^20^ leading to picosecond spectral signals in the visible and near-IR that reflect their nanostructural environment and trapping behavior.^19,20^ Motivated by this prior success, we hypothesized that TA measurements of photoexcited polarons can be integrated with spectroelectrochemistry (SEC), a technique called TA-SEC, to investigate the relationships between charge transport, ionic–electronic coupling, and voltage-dependent structural dynamics in working OMIECs. Traditional SEC simultaneously measures the electrochemical characteristics and optical absorbance of the ground electronic states of different redox species in a material.^22^ Alternatively, transient absorption spectroelectrochemistry (TA-SEC) couples measurements of the electrochemical behavior of a material with its excited state dynamics.^23^ Unlike absorbance spectroscopy, which measures doping-dependent optical transitions reflecting changes in the relative populations of polarons and neutral chain segments, TA-SEC exploits the sub-picosecond photophysics of polarons to directly link their transport behavior to OMIEC structure and the electronic–ionic environment.

In this work, we demonstrate TA-SEC as a powerful *in situ* technique for probing the electronic nature and location of charge carriers in a model polymer electrode. To test this method, we use a commonly studied and exceptionally stable high-performance polymer, poly(3,4 ethylenedioxythiophene):poly(styrenesulfonate) or PEDOT:PSS, as the model OMIEC system. PEDOT:PSS is an intrinsically doped material comprising a heterogeneous blend of a p-type semiconductor (PEDOT) and a nonconjugated polyelectrolyte (PSS).^7^ In thin films, the redox-mutable PEDOT chains pack together to form charge-conducting, PEDOT-rich domains that are embedded within an ion-conducting, PSS-rich matrix.^1^ When introduced into an aqueous electrolyte solution (*e.g.*, NaCl or KCl), the PSS-rich regions take up solvated ions, while the electronic transport of hole carriers (polarons) predominately occurs within the conjugated PEDOT framework. Because PEDOT:PSS is intrinsically conductive, devices containing this material operate in depletion mode, such that applying negative voltages reduces the PEDOT chains, depleting the carriers.

Herein, we present the first *in situ* TA-SEC measurement of an OMIEC polymer electrode in its working electrochemical environment using photoexcitation of polarons. Unlike a previous TA-SEC study, which excited the excitonic (bandgap) transition of neutral polymer chains in a ProDOT copolymer,^24^ exciting the charge carriers enables direct monitoring of their properties and behaviors. We begin by examining working PEDOT:PSS electrodes in an aqueous electrolyte using steady-state absorbance SEC measurements. A method is introduced for expanding the spectral window into the near-IR to enable measurement of the low-energy bands arising from polarons and bipolarons. Analysis of absorbance difference spectra as a function of applied voltage revealed new insights into the unexplored red shift of the high-energy P2 polaron band, which arises from the overlapping absorption of multiple polaron intermediates that interconvert based on the dynamic nanomorphology of PEDOT:PSS. Furthermore, the TA-SEC measurements revealed that deeply trapped polarons counterintuitively form at low doping levels (voltages), despite the general belief that polarons first enter the ordered, nanocrystalline PEDOT domains. At higher doping levels (voltages), the PEDOT:PSS nanomorphology and PEDOT packing structure evolve, decreasing polaron trapping. The results, which provide a clearer picture of the dynamic structure–property relationships in working PEDOT:PSS electrodes, lay the groundwork for future *in situ* TA-SEC studies of emerging polymers that will help guide the development of new high-performance OMIECs.

## Results and discussion

### Spectroelectrochemical cell design

Spectroelectrochemistry (SEC) is an effective method for studying the electrochemical doping processes occurring in organic mixed ionic–electronic conducting (OMIEC) polymer films.^22,25–27^ When applying an external bias in the presence of an external electrolyte, electronic charge carriers and ions diffuse into the OMIEC film, accumulate until reaching equilibrium with the external environment, and then begin mass transport across the electrolyte–OMIEC polymer electrode interface.^28^ This induces polymer swelling and additional structural dynamics including changes in polymer backbone planarity, π-stacking and arrangement of chains, and film morphology.^7^ At the molecular level, doping introduces a charge carrier along the conjugated polymer backbone that is charge-stabilized by an ion from the electrolyte. Simultaneous absorbance measurements in the visible and near-infrared (near-IR) regions provide spectral information^14,22^ about the electronic nature of the charged species (*e.g.* polarons, bipolarons) and their interconversions.

For SEC measurements, a three-electrode cell is typically constructed using a standard 1 cm cuvette, which holds a polymer-coated working electrode (WE), counter electrode (CE), and reference electrode (RE) in an electrolytic solution.^16^ However, when aqueous electrolytes are used, strong background absorbance by the water in the near-IR region practically limits the spectral range to approximately <1400 nm (or >0.88 eV).^22,25,26^ This not only prevents measurement of the lowest-energy electronic transitions of the charge carriers (*i.e.*, polarons and bipolarons), but also significantly restricts the choice of near-IR excitation wavelengths in TA spectroscopy to those that can photoexcite charge carriers without unintentionally exciting high-energy vibrational modes in the solvent (*i.e.*, <900 nm or >1.38 eV).

**Fig. 1:**
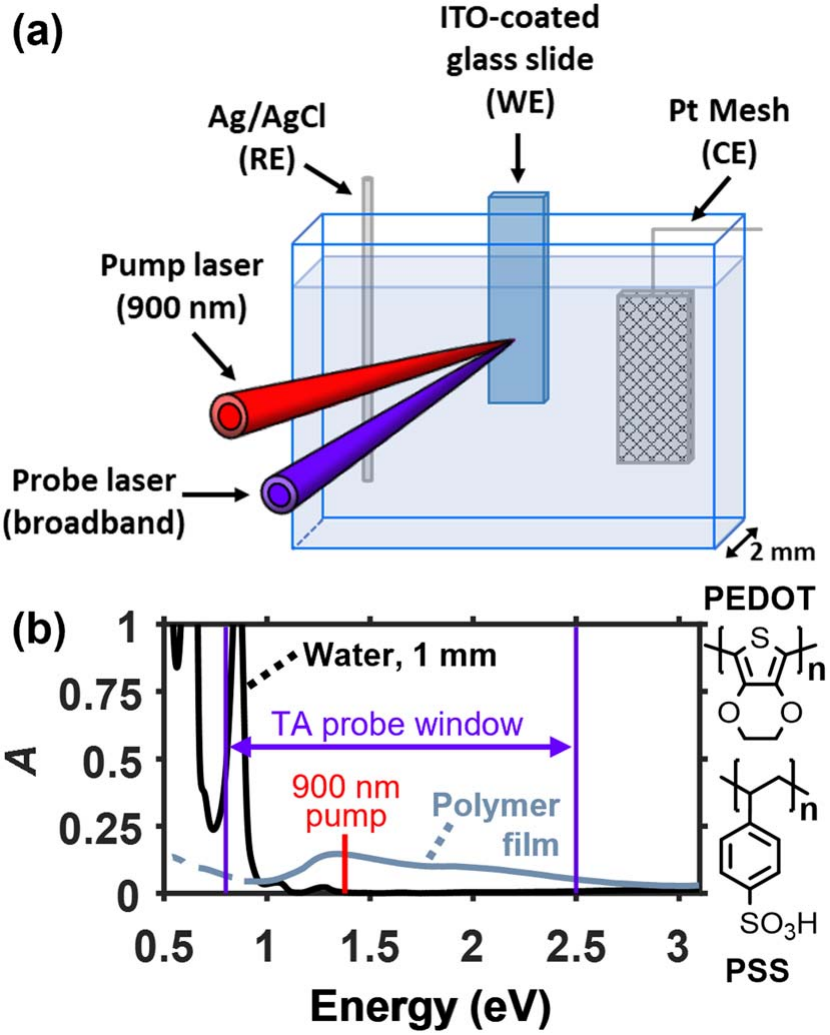
(a) Spectroelectrochemical cell for electrochemical doping of conducting polymers in an aqueous electrolyte designed for steady-state and ultrafast transient absorption spectroscopy in the visible/near-infrared region. (b) Absorption spectrum of a 1 mm layer of water compared to that of a doped PEDOT:PSS film on ITO/glass held at −0.2 V (*vs.* Ag/AgCl), highlighting the pump and probe wavelengths used in the TA measurements. The 900 nm pump wavelength is selected because it can excite the polaronic transition in the polymer without exciting a vibrational transition in the water. Immeasurable parts of the spectrum due to intense water absorption were removed.

To overcome these challenges, we designed an SEC cell using a wide colorimeter cuvette with a 2 mm path length that can easily fit all three electrodes while minimizing electrolyte absorbance (Fig. 1a). This cell is constructed using all commercially available parts that are widely accessible (see Section S1 of the SI), including a 2 mm enclosed Ag/AgCl RE and a platinum mesh CE. The WE consists of a polymer film spin-coated onto a semi-transparent indium-doped tin oxide (ITO) film electrode on a 0.7 mm-thick glass substrate. When placed in the cuvette, approximately 1 mm of electrolyte fills the space between the front window and the polymer film, which both minimizes the background absorption by water and reduces temporal broadening of the pump and probe pulses in TA spectroscopy.

Fig. 1b shows the absorption spectrum of a 1 mm layer of water along with the spectrum of a doped PEDOT:PSS film on ITO/glass in the SEC cell. Based on the spectrum of water, the spectral detection window for steady-state absorbance spectroscopy was determined to be 0.54–3.1 eV (400–2300 nm), where only 2 narrow regions of the spectrum must be removed due to intense background absorption. As illustrated below, the spectral window for ultrafast transient absorption (TA) measurements was 0.8–2.48 eV (500–1550 nm) using our probe supercontinuum source (see the purple arrow in Fig. 1b), although measurements extending to *ca.* 0.66 eV (1875 nm) are in principle possible. Widening the spectral window enables measurements of the high-energy side of the lowest energy polaronic band appearing below 1 eV, in addition to key transient absorption signals that report on polaron trapping (*vide infra*).

Prior to each absorbance and transient absorption measurement, the PEDOT:PSS films were “broken in” electrochemically using cyclic voltammetry (CV) (see Fig. S2.1 in the SI). This break-in process is generally understood to involve equilibration of the polymer during the early stages of swelling, where ion and electrolyte penetration induces initial changes in polymer morphology.^2,7^ We found that 3 cycles of CV over the electrochemical window of −0.9 V to +0.5 V were sufficient for PEDOT:PSS films treated with or without ethylene glycol. After the film is broken in, chronoamperometry (CA) is used to re-equilibrate the polymer at each selected voltage before spectroscopy measurements. From the CA data in Fig. S2.2, we found an equilibration time of 120 seconds to be sufficient (see the SI).

### Absorbance spectroelectrochemistry

**Fig. 2:**
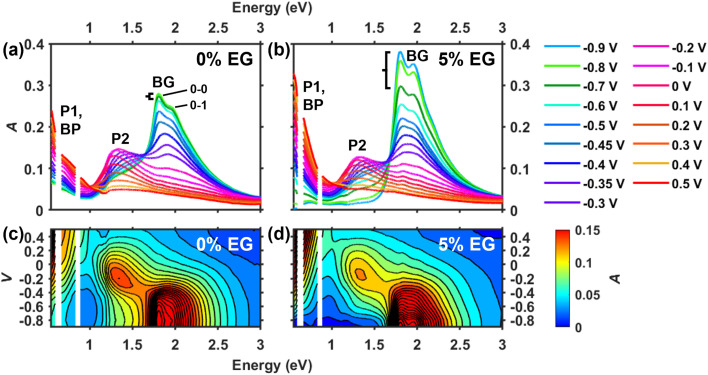
(a and b) Voltage-dependent absorption spectra of 0% EG and 5% EG PEDOT:PSS films in 0.5 M KCl. Each voltage (*vs.* Ag/AgCl) was applied for 120 s to equilibrate the film prior to recording the absorbance. The black brackets point out changes in the bandgap absorption signal when the voltage is changed from −0.9 to −0.7 V. (c and d) Contour plots of the absorbance data in (a) and (b) using 25 evenly spaced lines.

To guide the interpretation of the transient absorption results, steady-state absorbance SEC was first used to investigate the electrochemical doping processes and electronic nature of the charge carriers in PEDOT:PSS films as a function of fixed voltage (or doping level). Fig. 2a shows the voltage-dependent absorption spectra of a pristine PEDOT:PSS film (“0% EG sample”) in 0.5 M KCl electrolyte. Since PEDOT:PSS is a depletion mode polymer, applying negative voltages reduces (dedopes) the polymer to restore its neutral form, which is observed as an increase in the band gap (BG) absorbance near 1.8 eV, corresponding to a π–π* transition in neutral PEDOT chains. As the voltage is increased from −0.9 V to −0.1 V, the polymer becomes oxidized (doped), leading to a loss of the BG signal and a simultaneous increase in the P1 and P2 near-IR transitions arising from hole polarons (see Fig. 2a). The observation of a P2 band near 1.4 eV clearly indicates the formation of polarons in PEDOT chains. Interestingly, the P2 band gradually redshifts as voltage is increased to −0.1 V (Fig. 2a), a feature that remains poorly understood. The diagonally elongated feature near 1.4 eV in Fig. 2c highlights this shift. Continuing to increase the voltage from −0.1 V to +0.5 V results in a decrease in P2 band intensity and a simultaneous growth in absorbance below ∼1 eV, indicating the formation of electronically coupled polarons and/or spin-paired bipolarons (BPs) that typically occur at higher charge densities.^22^

Compared to the 0% EG sample, the film fabricated from a PEDOT:PSS solution containing 5% ethylene glycol (“5% EG sample”) showed similar overall changes in its absorption spectrum with increasing voltage (Fig. 2b). However, two key differences were seen: first, the BG absorption band exhibited a sharper vibronic structure at almost all voltages for the 5% EG sample (especially compare the traces at −0.9 V to −0.45 V in Fig. 2a and b), and with slight differences in the relative intensities of the 0–0 and 0–1 peaks. Second, the P1 and P2 absorption bands disappear completely for the 5% EG sample as voltage is decreased to −0.9 V; for the 0% EG sample, decreasing the voltage from −0.7 V to −0.9 V barely changes the absorption intensity of the P2 and BG bands (see black brackets in Fig. 2a and b). The 2D representation of the absorbance data in Fig. 2c and d additionally highlights the loss of the P1 and P2 absorption bands for the 5% EG sample. This can also be visualized by plotting absorbance against voltage for a series of wavelengths (see Fig. S3.1 in the SI).

The vibronic structure in the absorption spectrum of polythiophenes, which has been studied extensively,^25,27,29–31^ reflects the intra- and interchain excitonic interactions in the polymer film and therefore reveals information about nanoscale ordering and packing of the PEDOT chains. The absorption spectra of the 0% EG sample at the most negative voltages show clear vibronic structure with a relatively more intense 0–0 peak (Fig. 2a). This indicates strong intramolecular coupling between thiophene rings within PEDOT chains (J-aggregation), reflecting its tendency to planarize. This observation is consistent with prior reports,^32,33^ which argue that the ethylenedioxy groups attached to the thiophene rings facilitate planarization of the PEDOT chains by donating electrons into the conjugated system.^26,27,29^ For the 5% EG sample at the most negative voltages, the 0–1 vibronic peak is relatively more intense than that of the 0% EG sample (Fig. 2b), signifying an increase in intermolecular coupling between thiophene units in different polymer chains (H-aggregation). This enhancement of H-aggregate character is consistent with prior studies showing that EG improves PEDOT aggregation, leading to tighter π-stacking of the chains.^26,27,30,34^

The persistence of the P1 and P2 band intensities at the three most negative voltages for the 0% EG sample indicates incomplete reduction (dedoping) of the PEDOT chains, suggesting that not all polarons can be extracted from this film (Fig. 2a and c). Conversely, virtually all polarons are extracted in the 5% EG sample at the same applied potentials (Fig. 2b and d). While the slightly lower H-aggregate character of the PEDOT chains in the 0% EG sample is expected to raise its oxidation potential, this cannot explain the lack of change in absorption observed over the ∼200 mV range from −0.9 V to −0.7 V (see the black bracket in Fig. 2a). Therefore, we conclude that these polarons are not energetically trapped, but they must be spatially trapped within the film and unable to find a pathway to the electrode. These results are consistent with prior work by Rivnay *et al.*^26^ who showed how using EG as a co-solvent enhances the segregation of the PEDOT and PSS phases, resulting in a >10-fold improvement in hole mobility measured in a hydrated PEDOT:PSS film.

Next, we considered the origin of the voltage-induced redshift of the P2 band (see Fig. 2). It is generally believed that increasing the doping level in PEDOT:PSS^22,26,27^ or in other conjugated polymers^31,35,36^ shifts its coupled equilibria, which describe the interconversion between neutral and charged species,

1a n ⇌ p

1b p + p ⇌ bp

where n represents a neutral state, p is a polaron, and bp is a bipolaron. However, a shift of the P2 band could indicate either the presence of multiple polaron populations in different polymer phases, or a change in the electronic nature of the polarons that accompanies voltage-induced structural changes identified in PEDOT.^26,27^ We found that deeper analysis of the optical features using absorbance difference spectra, taken with respect to each voltage step, reveals a strikingly clearer picture of the doping process in PEDOT:PSS, including the identities of multiple polaron intermediates in contrast to eqn (1a) and (1b).

**Fig. 3:**
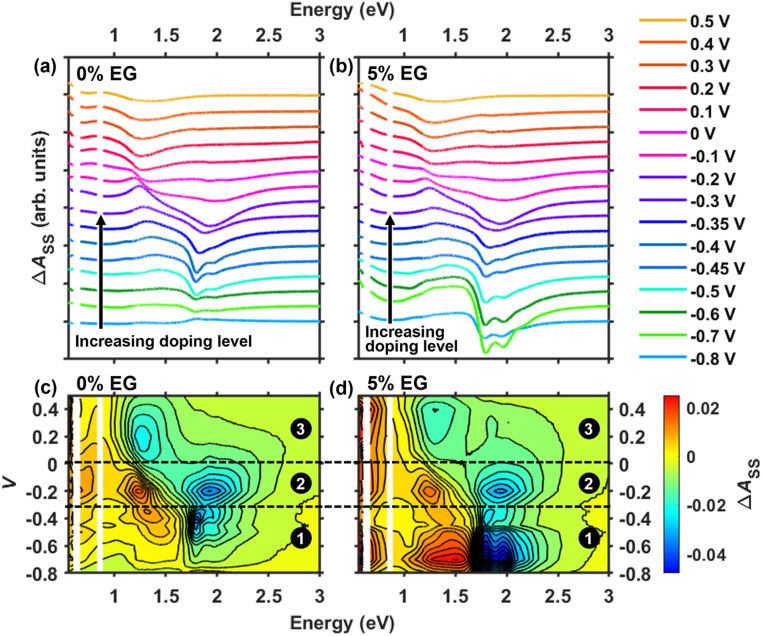
(a and b) Absorbance difference (Δ*A*_SS_) traces for the PEDOT:PSS films cast using 0% and 5% ethylene glycol (EG) cosolvent. The spectra, which are offset for clarity, were calculated by taking the difference in the absorption spectra with respect to each voltage step (*vs.* Ag/AgCl). (c and d) Contour plots of the absorbance difference data in (a) and (b) using 25 evenly spaced lines, where negative changes are in blue and positive changes are in red.

Fig. 3 shows the steady-state absorbance difference spectra (Δ*A*_SS_) for the 0% EG and 5% EG samples calculated from the data in Fig. 2 using

2 Δ*A*_SS_(*V*_*i*_) = *A*(*V*_*i*_) − *A*(*V*_*i*−1_)

where voltage is stepped from *V*_*i*−1_ to *V*_*i*_. Therefore, Δ*A*_SS_ reveals changes in the absorption spectrum due to each individual voltage step, while the spectra in Fig. 2 can only show cumulative changes in absorption during doping. Over each given voltage step, a negative Δ*A*_SS_ signal reflects an absorbing species that was depleted, whereas a positive Δ*A*_SS_ signal reflects absorbance of a new species that formed as a result. As will be discussed below, multiple overlapping signals can be distinguished from newly formed absorbing species during doping, such as polarons and bipolarons within different electronic environments or the presence of multiple polaron populations in different polymer phases.

Starting with low doping levels in the 0% EG sample, the Δ*A*_SS_ spectra show little change until voltage is stepped from −0.6 V to −0.5 V (compare dark green to light blue traces in Fig. 3a or see the bottom-half of region 1 in Fig. 3c). This reflects the poor extraction of spatially trapped polarons noted above. From approximately −0.6 V to −0.35 V, the negative BG band shows clear vibronic structure reminiscent of J-aggregates, while a positive P2 band forms near 1.5 eV (see Fig. 3a and the top-half of region 1 in Fig. 3c). This indicates that during the first stage of doping, oxidation occurs among planar PEDOT chains with high degrees of intrachain coupling. As we will discuss later, these are coulombically trapped interfacial polarons that likely form on the surfaces of PEDOT-rich domains.

At intermediate doping levels, an electronically distinct polaron population is observed in the 0% EG sample. When voltage is stepped from −0.35 V to −0.1 V, the negative BG band has a line shape reminiscent of disordered H-aggregates, while a positive, narrow P2 band appears at ∼1.25 eV (see purple and pink traces in Fig. 3a or region 2 in Fig. 3c). Additionally, a small negative P2 feature appears near 1.5 eV, indicating that some of the polarons that form at low doping levels are also depleted. To more clearly visualize the negative BG and P2 bands, see the normalized Δ*A*_SS_ spectra in Fig. S3.2 of the SI. Altogether, these observations indicate the doping of H-aggregated PEDOT chains with greater degrees of interchain coupling at intermediate doping levels, along with a depletion of the trapped polarons.

While PEDOT chains intrinsically form J-aggregates, the Δ*A*_SS_ spectra strikingly reveal the presence of a significant population of disordered H-aggregates that become oxidized at intermediate doping levels (*i.e.*, between −0.35 V and −0.1 V). This suggests that as voltage is increased, the PEDOT chains exhibit a greater degree of interchain coupling, possibly due to stronger π-stacking interactions. Contraction of π-stacked chains, which occurs during lamellar expansion, has been repeatedly observed in both chemically and electrochemically doped polyalkylthiophenes,^37–41^ and has also been indirectly inferred to occur in electrochemically doped PEDOT:PSS films near −0.2 V (*vs.* Ag/AgCl).^27^ Our *in situ* absorption spectroscopy results support these findings, and the impacts of this phenomenon will be addressed below using the transient absorption spectroelectrochemical results.

At high doping levels above −0.1 V, a negative P2 band near 1.25 eV and a positive band below ∼1.2 eV both appear, while there is virtually no change in absorption in the BG region around 2 eV (see the red to yellow traces in Fig. 3a or region 3 in Fig. 3c). This indicates the formation of bipolarons upon polaron fusion (*i.e.*, eqn (1b)), in which the densely arranged polarons interact and spin-pair.^35^ This is expected due to the neutral species being completely consumed at a higher level of doping and increased charge delocalization in the polymer film.^22,27^

Turning to the 5% EG sample, the changes in its Δ*A*_SS_ spectra largely mimic those of the 0% EG spectra, but with some differences (see Fig. 3b and d). First, there is significant depletion of the neutral PEDOT phase at the lowest voltages below −0.5 V (see Fig. 3b or the bottom-half of region 1 in Fig. 3c) because this film lacks spatially trapped polarons. Second, the negative P2 band seen at intermediate doping levels exhibits sharper vibronic structure (see Fig. S3.2 in the SI), indicating the presence of H-aggregates with a slightly higher degree of chain ordering. This is consistent with reports of a higher degree of crystallinity in PEDOT:PSS films accompanying phase segregation when EG cosolvent is used.^26,29,30,34^

In summary, we conclude that two electronically distinct polaron intermediates, which are gated by voltage-induced structural dynamics, form during electrochemical doping of PEDOT:PSS. The redshift in the P2 band seen in the voltage-dependent absorption spectra reflects the interconversion between these two polaron populations. Finally, we provided spectroscopic evidence for spatially trapped polarons in the pristine (0% EG) PEDOT:PSS film that cannot be extracted due to limited percolation pathways, and not likely due to the slight differences we observed in the PEDOT chain packing.

### Ultrafast transient absorption spectroelectrochemistry

Herein, we describe the first transient absorption (TA) spectroelectrochemical (TA-SEC) measurements of a conducting polymer using direct photoexcitation of the charge carriers. While the steady-state absorbance SEC results above provided information about polymer chain structure and the interconversion of the polaronic species in PEDOT:PSS, direct transient absorption measurements of polarons can reveal information about both their nanoscale environment^20^ and local mobility.^19,42^ The results show how *in situ* TA-SEC measurements, which are sensitive to ionic–electronic coupling and voltage-induced structural dynamics, can be used in combination with traditional SEC to more clearly investigate structure–property relationships^1,2,16^ and nanoscale charge transport (or trapping) in OMIEC polymers.

In the TA-SEC measurement, the polarons created at each fixed voltage are first photoexcited *via* the P2 transition by a narrowband ∼200 fs pump pulse, which induces spatial separation of the ion–polaron pairs *via* charge transfer with a nearby coupled neutral chain segment.^43^ Then the picosecond changes in absorbance in the visible and near-IR regions are recorded using a broadband probe pulse to measure the polaron relaxation and ion–polaron recombination dynamics. Because the latter process is diffusion-limited, the decay kinetics of the TA signals provide information about their local mobilities.^19,42^ At the same time the local transient electric field, which intensifies due to ion–polaron separation, perturbs the BG absorption of nearby neutral chain segments, revealing details of their nanoscale environment.^20^ We found that care must be taken to avoid unintentional excitation of solvent vibrational modes, which leads to overlapping features in the TA spectrum that obfuscate signals from the polymer. Therefore, we chose an excitation wavelength of 900 nm (1.38 eV), which selectively excites the P2 transition over the high-energy vibrational mode of water appearing at 976 nm (1.27 eV) (see Fig. 1b).

**Fig. 4:**
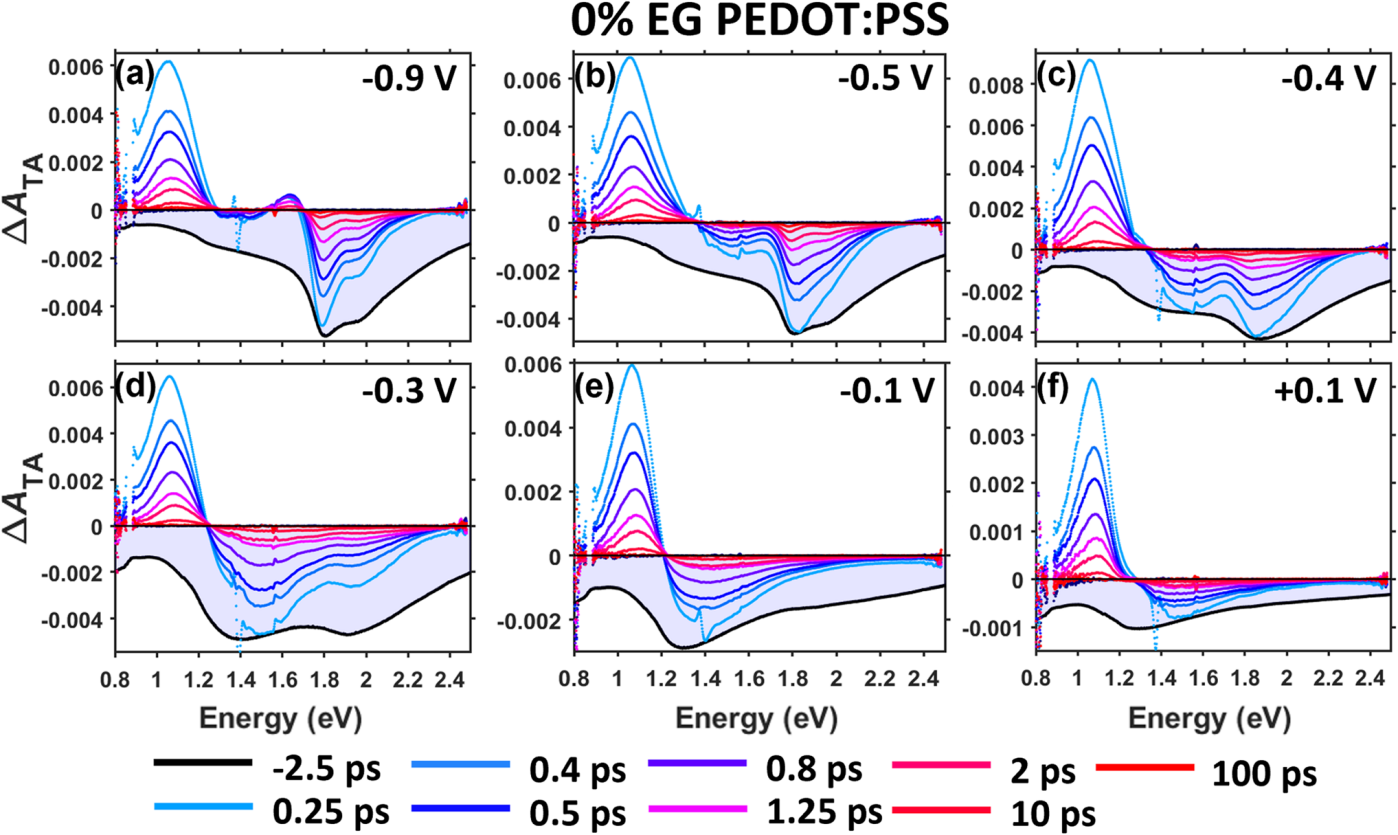
TA-SEC measurements of the pristine PEDOT:PSS film in 0.5 M KCl electrolyte in the visible and near-IR spectral regions as a function of applied voltage (*vs.* Ag/AgCl). The voltages used were (a) −0.9 V, (b) −0.5 V, (c) −0.4 V, (d) −0.3 V, (e) −0.1 V, and (f) +0.1 V, and the excitation energy was 1.37 eV (900 nm). Prior to each measurement, the film was allowed to equilibrate under bias for 120 seconds. The associated ground state absorbance recorded at each voltage was inverted, arbitrarily scaled, and overlaid with the TA spectra to guide interpretation of the Δ*A*_TA_ spectra (light blue shaded regions).

Fig. 4 shows the TA spectra recorded for the pristine PEDOT:PSS film (0% EG sample) at a series of pump–probe time delays and as a function of applied voltage. Starting with −0.9 V, three primary TA features are seen: first, an electroabsorption (EA) signal^20^ appears in the BG region, featuring a positive band at ∼1.64 eV and a negative band with vibronic structure around 1.88 eV resembling that of J-aggregates (Fig. 4a). The formation of an EA signal within 0.25 ps indicates rapid polaron–ion photodissociation, which occurs within our ∼200 fs time resolution. Note that this EA signal overlaps with a ground state bleach (GSB) of the BG transition accompanying charge transfer of the polaron. Second, a ground state bleach (GSB) of the P2 band appears at ∼1.4 eV due to depletion of the electronic ground state of the polaron. Third, a photoinduced absorption (PIA) band appears at ∼1.05 eV arising from both polarons in the electronic excited state and polarons that have thermalized to locations where they have greater ion–hole distances than in the ground state.

**Fig. 5:**
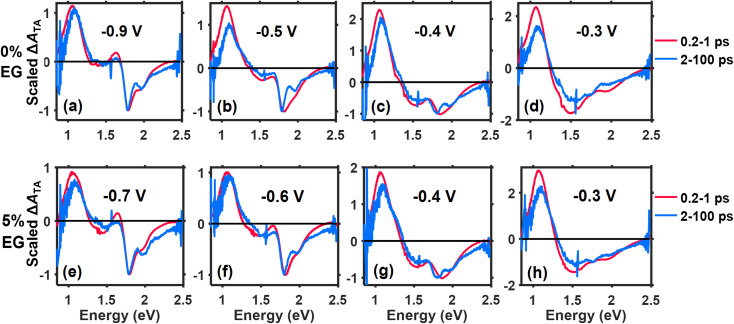
Voltage-dependent transient absorption spectra of PEDOT:PSS films in 0.5 M KCl (*vs.* Ag/AgCl) averaged over early (0.2–1 ps) and late (2–100 ps) time windows and scaled to the 0–0 vibronic peak of the BG transition for the (a–d) 0% EG and (e–h) 5% EG samples. Voltages were chosen such that similar doping levels could be compared between the 0% EG and 5% EG samples.

As voltage is increased from −0.9 V to −0.3 V (increase in doping level), the intensity of the BG signals (*i.e.*, EA + bleach) decreases relative to that of the P2 bleach (Fig. 4b–d). Over this voltage range, the positive part of the EA signal, which spectrally overlaps the P2 bleach peak, becomes weaker as the polaron density increases (also see Fig. 5). The BG bleach also decreases in intensity upon increasing voltage (doping level) to −0.3 V because the number of neutral chain segments becomes more and more depleted. Simultaneously, the vibronic structure in the BG band seen at the earliest time delays washes away, and the overall line shape evolves into one resembling that of disordered H-aggregates, consistent with observations from the steady-state absorbance difference spectra (Fig. 3). At the highest doping levels (*i.e.*, −0.1 V and +0.1 V), the BG signals become relatively very weak and only the P2 bleach and PIA bands are observed. The TA-SEC spectrum of the 5% EG sample shows the same features and behaviors as that of the 0% EG sample (see Fig. S4.1 in the SI).

The decay of all TA signals in the 0% EG sample (Figure 4) and 5% EG sample (Fig. S4.1) mostly occurs within the first picosecond, followed by a slower decay into the late picosecond timescale. This biphasic decay behavior has been consistently seen in TA spectroscopy studies of chemically doped polythiophenes by our group^19,20,44^ and others,^42,43^ and is due to overlapping signals from coulombically-free and trapped polarons with different characteristic decay rates. For coulombically-free polarons, or polarons that are not electrostatically bound to counterions, photoexcitation creates an electronic excited state that very rapidly relaxes (thermalizes) to restore the electronic ground state within 100s of femtoseconds.^42^ For coulombically-trapped polarons, or polarons that are bound to counter ions, the electronic excited state first thermalizes to a location farther away from its counterion, followed by slower diffusion-mediated recombination of the polaron and ion to restore their ground state distance.^42^ Therefore, the TA spectrum at early time delays (*e.g.*, ≤1 ps) mostly reflects the spectrum of free polarons (if they are present), whereas the TA spectrum at later time delays (*e.g.*, >1 ps) clearly reflects that of polarons which remain transiently separated from their counterions.

In the first TA study^45^ of a PEDOT:PSS film, Meskers *et al.* investigated the ultrafast dynamics of charge carriers excited with 1.2 eV infrared light. The authors reported rapid decay of the transient signals on the early picosecond timescale, which was assigned to a thermal effect arising after <250 fs electron–electron thermalization based on charge-induced absorption and photo-induced absorption measurements. However, we note that the full time-spectrum TA signal could not be measured, and their TA spectral window was limited, making a clear assignment difficult. Furthermore, the PEDOT:PSS film was heavily doped and did not show a clear P2 absorption band, suggesting that bipolarons were predominantly photoexcited in their measurement. In contrast, recent studies on conducting polymers doped to a lower degree have consistently shown that the decay of photoexcited polarons leads to spectral features more consistent with a transiently separated ion–charge pair.^19,20,42,43^

Fig. 5 shows the voltage-dependent TA spectra of the PEDOT:PSS films averaged over 0.2–1 ps (“early time”) and 2–100 ps (“late time”) that have been scaled to the 0–0 vibronic peak at 1.79 eV. Note that the voltages for comparing the 0% EG and 5% EG samples were selected such that they correspond to similar doping levels of the polymer (*e.g.*, compare −0.9 V for the 0% EG sample to −0.7 V for the 5% EG sample). Note also that Fig. 5 compares the TA spectra for the lowest 4 doping levels, which exhibit moderate-to-strong BG signals, while the spectra for the highest 2 doping levels lack detectable BG signals and are plotted in Fig. S4.2 (see SI).

For both the 0% EG and 5% EG samples, the BG bands in the early and late TA spectra at the two most negative voltages (lowest two doping levels) exhibit clear vibronic peaks with a relatively more intense 0–0 peak (Fig. 5a, b, e and f). This indicates that the polarons reside in regions of the PEDOT phase containing planar chains with strong intrachain coupling (J-aggregate character), consistent with the results from steady-state SEC above. The vibronic structure is less sharp at early times, which we tentatively assign to inhomogeneous broadening, suggesting that the free polarons reside in wet, nonpolar PEDOT environments with correspondingly weak ion–polaron interactions. On the other hand, the sharpened vibronic structure seen at later times suggests that the trapped polarons reside in the nonpolar regions of the polymer with stronger ion–polaron interactions.

These results suggest that although electrochemical doping initially yields polarons in ordered regions of the PEDOT phase, many of them are counterintuitively trapped. The P2 band of these polarons contains vibronic structure for both the 0% EG and 5% EG samples (see the absorbance difference spectra from −0.8 V to −0.5 V in Fig. 3 and S3.2 in the SI). In an electrochemically doped oligoether-functionalized 3,4-propylenedioxythiophene (ProDOT) copolymer, Bargigia *et al.* associated such vibronic features with electronic states having charge-transfer (CT) character, which form at low voltages prior to polaron formation.^24^ We therefore assign the trapped polarons that form in PEDOT:PSS at low voltages to deeply trapped polarons with strong CT character. We postulate that these polarons reside at the surfaces of the densely packed, PEDOT-rich domains where they can strongly interact with PSS^−^ counterions; the weaker or missing interchain coupling in these J-aggregated domains could prevent the polaron from escaping away from PSS^−^. A similar phenomenon has been reported in electrochemically doped poly(3-hexylthiophene) films, in which polaron–ion pairs form at the interfaces between ordered and disordered domains at low voltages.^14,37,46^

At intermediate doping levels (*e.g.*, −0.4 V and −0.3 V), the BG band in the TA spectrum at early times evolves into one reflecting H-aggregation (see red traces in Fig. 5c, d, g and h), consistent with changes seen in the absorbance difference spectra above (Fig. 3). This suggests that this polaron population, which forms within disordered H-aggregates of PEDOT produced by doping-induced lamellar expansion, does not interact strongly with PSS^−^ counterions. Based on these results, we further posit that the stronger interchain interactions present in these H-aggregates of PEDOT, combined with the uptake of electrolyte during lamellar expansion, increase the relative population of free (mobile) polarons in PEDOT:PSS.

**Fig. 6:**
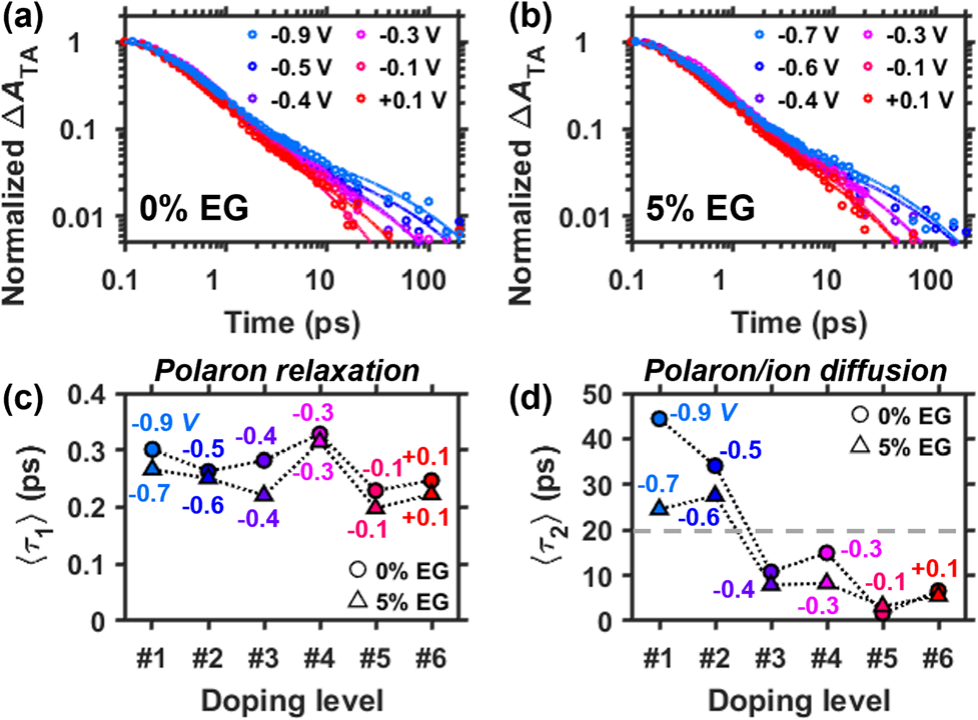
Voltage-dependent (*vs.* Ag/AgCl), normalized TA kinetics selected at the PIA peak maximum and averaged over 1.05–1.09 eV for the (a) 0% and (b) 5% EG samples. Data are displayed as markers, while the solid lines are fits to a sum of two stretched exponentials as described in the text. Average lifetimes of the (c) short-lived (〈*τ*_1_〉) and (d) long-lived (〈*τ*_2_〉) photoexcited polaron populations in the 0% and 5% EG samples. The average lifetimes were calculated using the fit parameters from the stretched exponential modeling of the kinetic data in (a and b).

Next, we investigate the voltage-dependent TA decay kinetics of the PEDOT:PSS films. As shown in our prior studies of chemically doped polythiophenes,^19–21^ the decay kinetics of the PIA peak of photoexcited polarons are sensitive to the degree of trapping of the charge carriers. Fig. 6a and b shows the normalized decay kinetics of the PIA band taken at the peak maximum for the 0% EG and 5% EG samples. In both samples, biphasic kinetics are observed, with the fast component decaying within the first picosecond and the slow component decaying over the next ∼10 to 100 picoseconds.

To quantify this kinetic behavior, we fit the TA kinetic data (markers in Fig. 6a and b) to the sum of two stretched exponentials convoluted with a Gaussian instrument response function (solid curves in Fig. 6a and b), as in our prior work^19^ and as further detailed in Section S5 of the SI. In this kinetic model, the first stretched exponential captures the electronic relaxation of the free and trapped polarons, while the second stretched exponential captures diffusion-mediated recombination of the ion–polaron pair. The average relaxation times of the fast (〈*τ*_1_〉) and slow (〈*τ*_2_〉) decay components, which are calculated from the stretched exponential fit parameters (included in Tables S5.1 and S5.2 in the SI), are plotted against the doping level for both the 0% EG and 5% EG samples in Fig. 6c and d.

Only the slow decay component, which reflects diffusion of the trapped polaron population, shows a dependence on voltage for both the 0% EG and 5% EG samples (see Fig. 6c and d). As voltage (or doping level) increases, the average lifetime of the slow component 〈*τ*_2_〉 generally decreases, indicating that the polarons are on average less trapped (more mobile) at higher doping levels. This observation is consistent with charge transport models, including the semi-localized transport^47^ and Kang–Snyder^48^ models, which suggest that charge mobility in conducting polymers increases at higher carrier densities. However, two more notable features in Fig. 6d are also seen: first, the polaron average relaxation time significantly drops between the second and third doping levels, when there are significant structural changes in the PEDOT phase from J- to H-aggregates accompanying lamellar expansion (highlighted by the dashed gray line). This correlation suggests that an increase in interchain coupling along with the uptake of electrolyte prevents the formation of deeply trapped polarons. The resulting enhancement of polaron delocalization and coulombic screening is expected to weaken electrostatic interactions of the ion–polaron pairs, which are known to significantly control trapping in chemically doped polymer films.^10,11,49^ Second, the 5% EG sample shows shorter polaron average relaxation times, especially for the first two doping levels, when there is a significant population of trapped polarons in J-aggregated chains. Tighter π-stacking of the PEDOT chains in the 5% EG sample, as discussed above, is expected to increase interchain delocalization of the polaron, subsequently weakening its interaction with a PSS^−^ counterion.

**Fig. 7:**
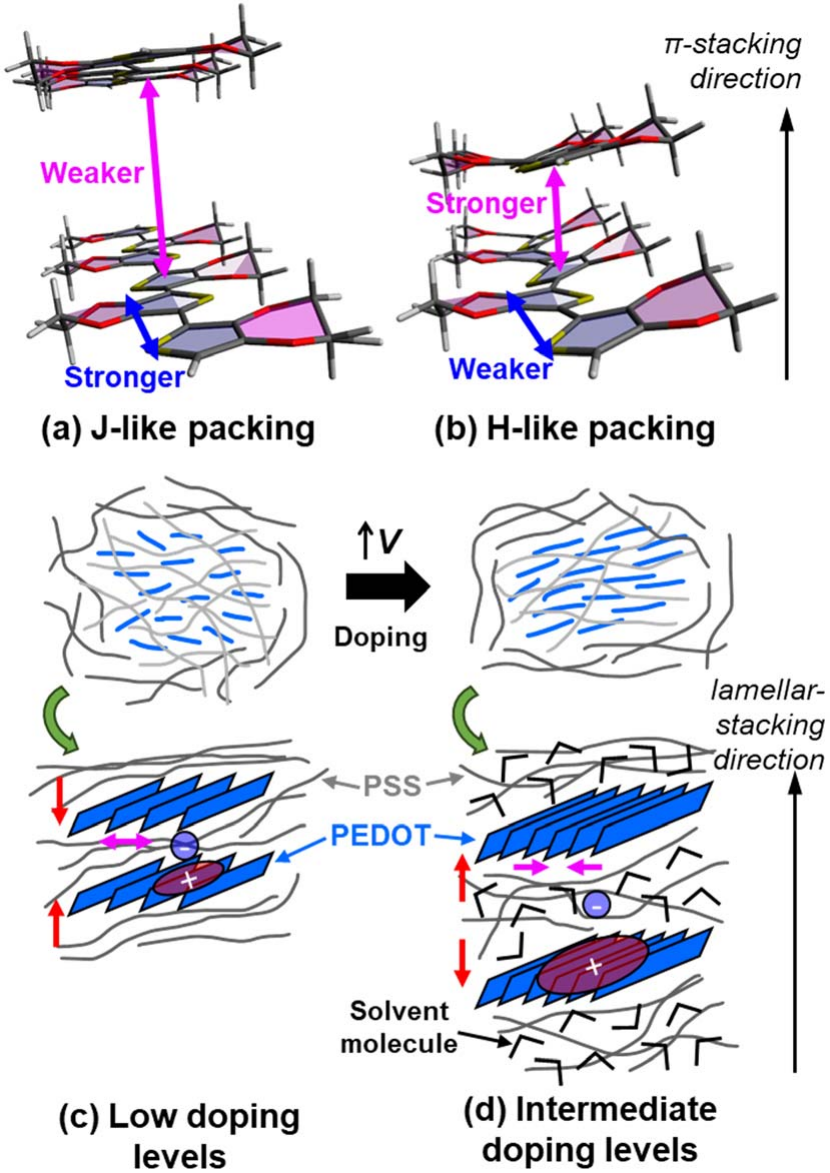
Illustration of the doping-induced changes in (a and b) PEDOT aggregation behavior and (c and d) PEDOT:PSS film nanostructure. In J-like aggregates (a), the PEDOT chains are more planar and have stronger intrachain coupling. The interchain coupling is weak and the chains are more isolated. In H-aggregates (b), the PEDOT chains are more twisted and can thus more tightly π-stack, increasing interchain coupling and decreasing intrachain coupling. At low doping levels (c), the lamellae are more compressed and the PEDOT chains are more separated like in J-aggregates with localized polarons. At intermediate doping levels (d), the lamellae expand with solvent from the electrolyte and the PEDOT chains pack more tightly like in H-aggregates with more delocalized polarons.

Based on the steady-state SEC and TA-SEC results, we conclude that multiple polaronic species form during the electrochemical doping of PEDOT:PSS that differ in their degree of trapping based on variations in the dynamic film nanostructure and aggregation of PEDOT chains (see Fig. 7). At low doping levels, deeply trapped polarons with CT character form among J-aggregated PEDOT chains in proximity to PSS^−^, where ion–polaron interactions are strong. Using EG cosolvent not only improves charge mobility by creating more percolation pathways *via* PEDOT/PSS phase segregation, but it also reduces polaron trapping by slightly enhancing interchain coupling in the J-aggregated PEDOT domains. At intermediate doping levels, lamellar expansion increases interchain (H-like) coupling and electrolyte uptake in the PEDOT domains, which reduces polaron trapping. At the highest doping levels, mostly free polarons are observed, as expected when the carrier density is high.

## Conclusions

The steady-state absorbance SEC and TA-SEC results together provided a clearer picture of the electrochemical doping processes occurring in PEDOT:PSS and how they are controlled by voltage-induced nanostructural dynamics. Steady-state SEC reveals structure-sensitive information about the polaronic species and their interconversions, while TA-SEC probes the local mobilities of polaron/ion pairs and their nanoscopic environment through analysis of the polaron–ion recombination kinetics and the vibronic structure of the BG bleach, respectively. In this work, we showed for the first time how combining these methods creates a powerful approach for the *in situ* probing of charge carriers during electrochemical charging of conducting polymer films. We further expect that this TA-SEC method can be applied more broadly to uncover structure–function relationships in accumulation mode and n-type polymers.

Our results also highlight the practical benefits of using smaller path length cells for SEC measurements, which can be constructed using widely accessible components. Expanding the SEC spectral window enables the measurement of important polaron and bipolaron signals in the absorption spectra that are often missed using traditional 1 cm path length SEC cells. Furthermore, we showed the importance of analyzing SEC absorbance difference spectra to provide deeper insights into the doping mechanisms. We hope that our study encourages others to more routinely implement these approaches when studying polymer doping.

## Author contributions

C. C.: conceptualization (equal); formal analysis (lead); methodology (equal); validation (lead); visualization (lead); writing/original draft preparation (lead); writing/review & editing (equal). A. R. U.: formal analysis (supporting); validation (supporting); visualization (supporting); writing/review & editing (supporting). C. G.: conceptualization (equal); formal analysis (supporting); funding acquisition (lead); methodology (equal); supervision (lead); writing/review & editing (equal).

## Conflicts of interest

There are no conflicts of interest to declare.

## Supplementary Material

SC-016-D5SC04619J-s001

SC-016-D5SC04619J-s002

## Data Availability

Supplementary information: Experimental methods and additional spectroscopic and electrochemical results are described in the SI. The spectroscopic and electrochemical data appearing in this article are available as text files, which have been included as part of the SI. See DOI: https://doi.org/10.1039/d5sc04619j.
