# Supplementary material for: In situ monitoring of polarons in a mixed conducting polymer using ultrafast transient absorption spectroelectrochemistry
Source: Chem Sci. 2025 Aug 27;16(38):17839–49. doi: 10.1039/d5sc04619j (PMC12412052; doi:10.1039/d5sc04619j)
Supplement: SC-016-D5SC04619J-s002 [file SC-016-D5SC04619J-s002.pdf]

Supplementary Information (SI) for Chemical Science.
This journal is © The Royal Society of Chemistry 2025

Supplementary Material for

In situ monitoring of polarons in a mixed conducting polymer using ultrafast transient absorption spectroelectrochemistry

Caitlyn Clark, Abdul Rashid Umar, Christopher Grieco

Dept. of Chemistry and Biochemistry, Auburn University, Auburn, Alabama 36849, USA

S1. Experimental methods 2
S2. Electrochemistry measurements 4
S3. Additional absorption spectroscopy results 5
S4. Additional transient absorption spectroscopy results 7
S5. Kinetic modeling of the transient absorption data 9
S6. References 14

Section S1. Experimental methods

Polymer film preparation

A 350 µL aliquot of a PEDOT:PSS aqueous suspension (Clevios PH 1000, Heraeus) was spin coated on a 40 mm x 10 mm x 0.7 mm, R_s= 25-35 Ω, ITO-covered glass slide (Delta technologies) at 2500 rpm for 90 s. The ITO-glass slides were pre-cleaned using acetone, isopropanol, and distilled water in a sonicator bath before activation with UV/O_3 for 10 min (Model 18, Jelight). After deposition, the polymer film was annealed at 140 ^oC for 45 min. To prepare the second sample, a 5% (v/v) ethylene glycol (EG) PEDOT:PSS mixture was prepared by adding 50 µL of EG to 950 µL PEDOT:PSS aqueous suspension, which was then sonicated for 3 min. After sonication, a 350 µL aliquot of the 5% EG PEDOT:PSS mixture was spin coated on an ITO-covered glass slide using the same procedure as for the 0% EG PEDOT:PSS film.

Steady-state absorption spectroelectrochemistry

A homebuilt absorbance spectrophotometer was used to record steady-state visible and near-infrared (near-IR) absorption spectra of the ground state of the polymer film in the 400 – 2500 nm (0.5 – 3.1 eV) spectral region, as previously described.[1–3] The spectroelectrochemical cell was comprised of a 2 mm path length Type 93 glass colorimeter cuvette (FireflySci, 93G2), a Ag/AgCl reference electrode (eDAQ, ET072-1), a platinum flag counter electrode (VWR), and an ITO-glass working electrode (Delta Technologies). The working electrode with the deposited polymer film, reference electrode, and counter electrode were immersed in 0.5 M potassium chloride, KCl, (J.T. Baker) as the aqueous electrolyte.

Chronoamperometry was used to apply a constant voltage by a WaveNow wireless potentiostat/galvanostat (Pine Research). Once the polymer's absorbance reached an equilibrium value (~2 min) under each applied voltage, a steady-state absorption spectrum was recorded with a <1 s acquisition time. Because water absorbs throughout the near-IR region (i.e., peaking at 1400 and 1950 nm), absorbance data were omitted in those spectral regions, where not enough light reached the detector.

Ultrafast transient absorption spectroelectrochemistry

Two different homebuilt ultrafast pump-probe spectrometers were used to perform broadband transient absorption (TA) spectroscopy. One TA system has spectral sensitivity in the visible/near-IR region (~1.24 – 2.48 eV), while the other has spectral sensitivity in the near-IR and short-wave IR regions (~0.53 – 1.38 eV). The instrument setups are discussed extensively in our previous works,[1-2] except that a 2350 nm pulse was used to drive white light supercontinuum generation for the probe beam for the latter TA system. The TA measurements were coupled with the previously discussed spectroelectrochemical cell setup, and voltages were applied using chronoamperometry for the ultrafast TA spectroelectrochemistry measurements. Before each TA measurement, the polymer film was allowed to reach electrochemical equilibrium for each voltage applied, which took approximately 2 min. The film was optically excited using 900 nm (1.38 eV) at a repetition rate of 2.5 kHz and with an excitation fluence of approximately 200 $\mu J/cm^2$. The TA signals were detected at the magic angle pump/probe geometry, and each ultrafast TA spectroelectrochemistry measurement took approximately 23 min, including the 2 min period to reach electrochemical equilibrium.

Section S2. Electrochemistry measurements

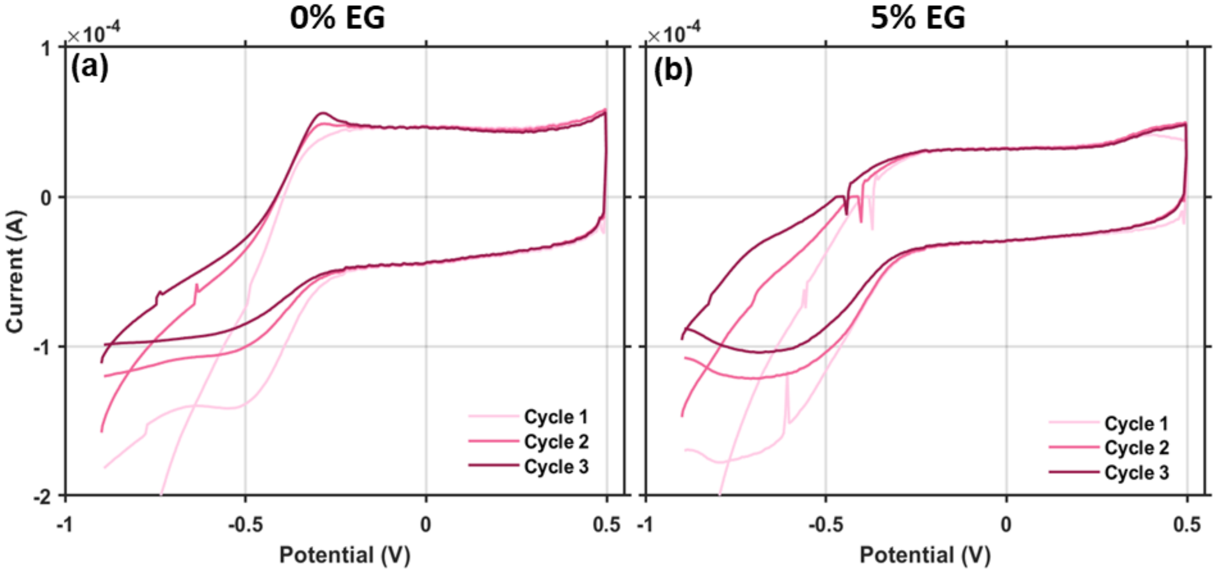

Figure S2.1. Cylic voltammograms of **(a)** 0% and **(b)** 5% EG samples over three cycles sweeping in the forward direction from -0.9 V to +0.5 V (vs Ag/AgCl) at a sweep rate of 25 mV/s.

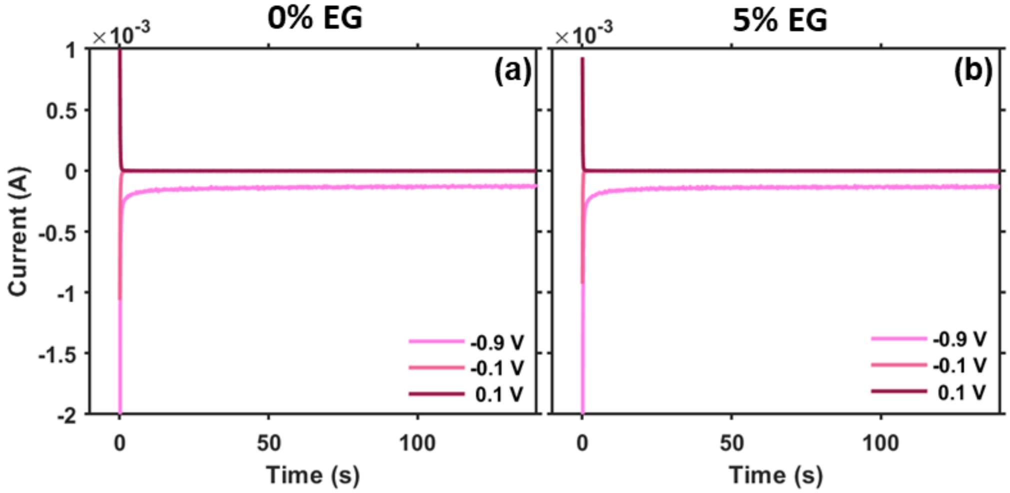

Figure S2.2. Chronoamperograms of **(a)** 0% and **(b)** 5% EG samples, which show when each polymer film reaches electrochemical equilibrium at different fixed applied voltages (vs Ag/AgCl) corresponding to dedoped (-0.9 V), moderately doped (-0.1 V), and highly doped (0.1 V) levels.

Section S3. Additional absorption spectroscopy results

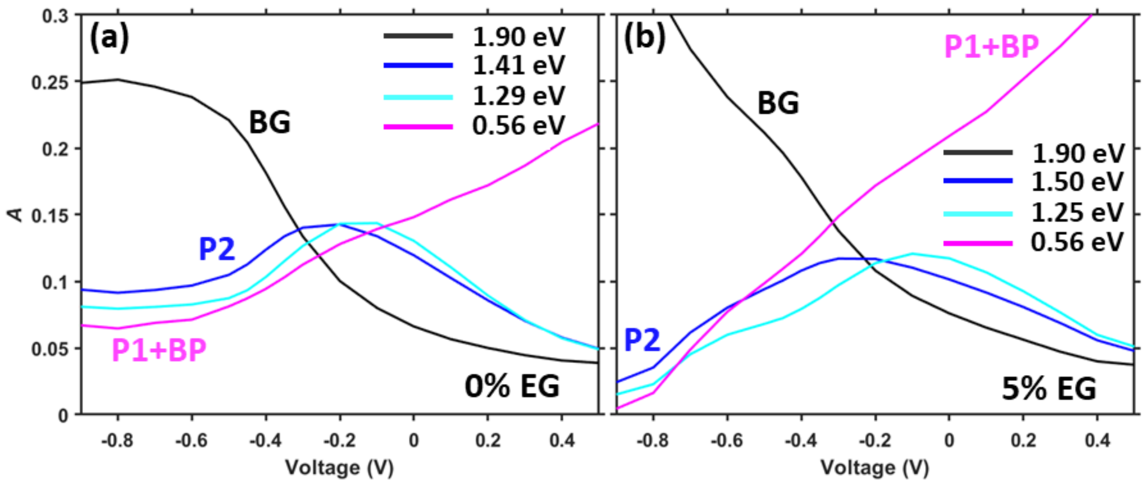

Figure S3.1. Absorbance versus voltage traces at energies corresponding to the neutral (BG), polaron (P1 and P2), and bipolaron (BP) transitions for **(a)** 0% EG PEDOT:PSS and **(b)** 5% EG PEDOT:PSS. The blue and cyan traces correspond to the absorbance at the blue and red edges of the P2 band, respectively.

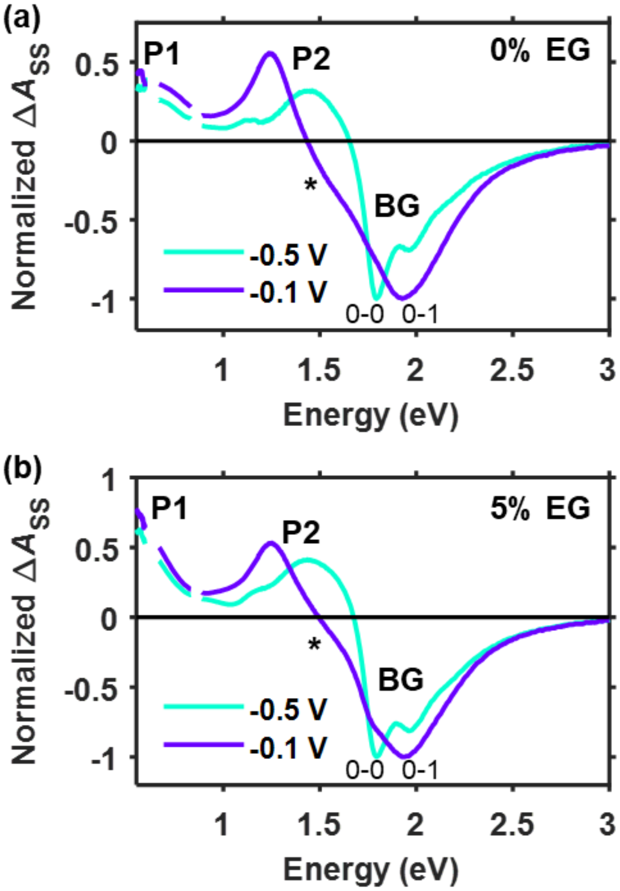

Figure S3.2. Comparison of the absorbance difference spectra taken at -0.5 V and -0.1 V for the **(a)** 0% EG and (**b)** 5% EG PEDOT:PSS film samples. The spectra are normalized to the bleached BG band. The P2 band for both films has vibronic structure at -0.5 V. At -0.1 V, the P2 band redshifts, leaving behind a P2 bleach feature near 1.5 eV (marked as *). The vibronic features in the BG bleach band significantly differ between -0.5 V and -0.1 V. Note that the 0-0 and 0-1 vibronic features in the -0.1 V spectra are more pronounced for the 5% EG sample.

Section S4. Additional transient absorption spectroscopy results

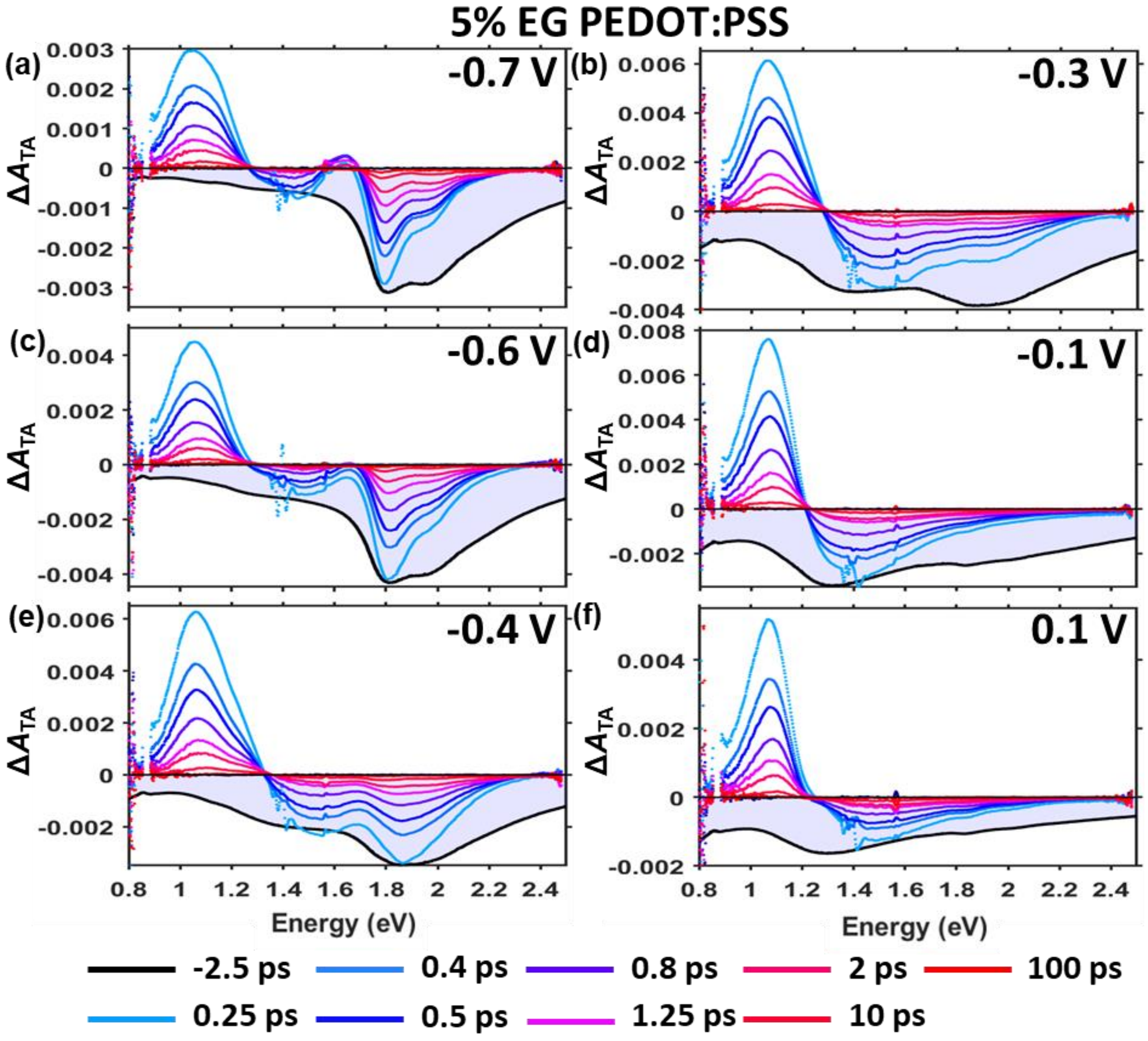

Figure S4.1. TA-SEC measurements of the 5% EG PEDOT:PSS film in 0.5 M KCl electrolyte in the visible and near-IR spectral regions as a function of applied voltage (vs Ag/AgCl). The excitation energy was 1.37 eV (900 nm). Prior to each measurement, the film was allowed to equilibrate under bias for 120 seconds. The associated ground state absorbance recorded at each voltage was inverted, arbitrarily scaled, and overlaid with the TA spectra to guide interpretation of the ΔA_{TA} spectra (light blue shaded regions).

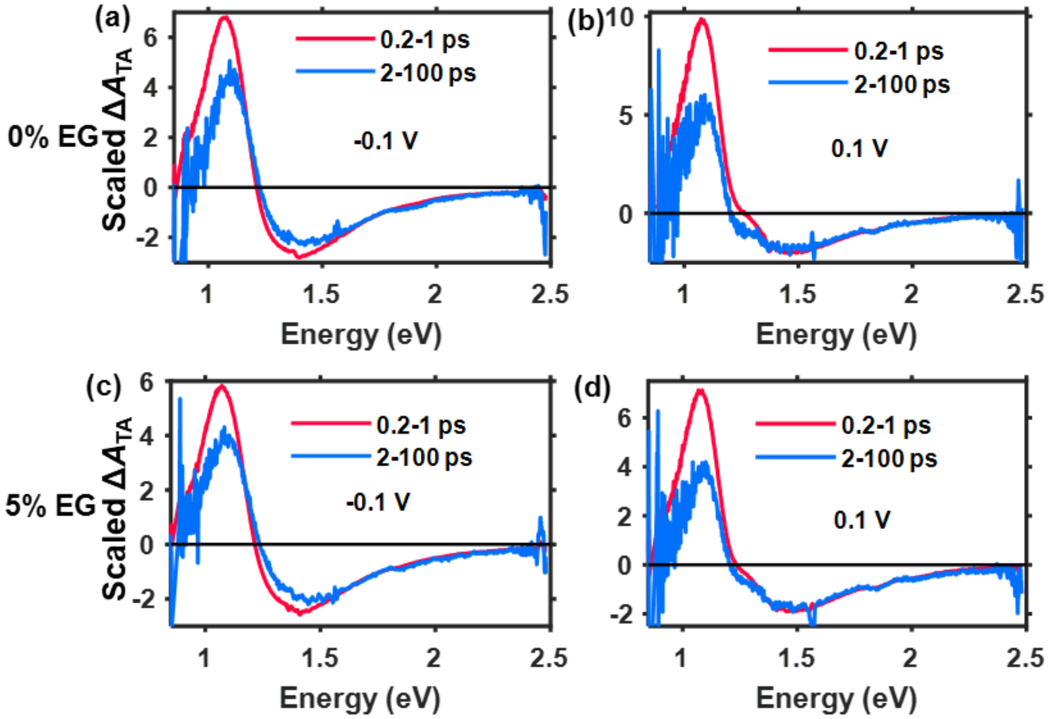

Figure S4.2. Voltage-dependent transient absorption spectra of PEDOT:PSS films in 0.5 M KCl (vs Ag/AgCl) averaged over early (0.2–1 ps) and late (2–100 ps) time windows and scaled to the 0-0 vibronic peak of the BG transition for the **(a,b)** 0% EG and **(c,d)** 5% EG samples. The applied voltage is indicated in each plot.

Section S5. Kinetic modeling of the transient absorption data

Due to their inherent disorder, polymer systems often exhibit dynamics that are well-described using empirical stretched exponential models, including for both structural relaxation and charge transport.[3] As described in the main text, we fit the normalized TA kinetic data taken at the peak of the photoinduced absorption (PIA) band, which captures both the fast and slow decay components, using a sum of 2 stretched exponentials convoluted by a Gaussian instrument response function (IRF),[4]

$$f(t) = \begin{cases} N\left(w_1 e^{-\left(t/\tau_1\right)^{\beta_1}} + (1-w_1)e^{-\left(t/\tau_2\right)^{\beta_2}}\right), & t > 0 \\ 0, & t \le 0 \end{cases}, \tag{1a}$$

$$\mathrm{IRF}(t) = ne^{-\frac{1}{2}\left(\frac{t}{(\mathrm{FWHM}/2.35482)}\right)^2}, \tag{1b}$$

$$\Delta A_{\mathrm{fit}} = \mathrm{IRF}(t) * f(t). \tag{1c}$$

where N is a normalization constant, w_1 is a positive weighting constant with $0 \le w_1 \le 1$, τ_1 and τ_2 are time constants, β_1 and β_2 are the William-Watts stretching parameters, t is delay time, $\mathrm{IRF}(t)$ is a Gaussian instrument response function, n is a normalization constant chosen such that $\int_{-\infty}^{\infty} \mathrm{IRF}(t)dt = 1$, and FWHM is the full-width at half-maximum. The area-normalized Gaussian function in eqn. 1b is centered at $t = 0$, and its FWHM represents the cross-correlation time of the pump and probe pulses in the TA measurement. We found a FWHM value of 0.18 ps to adequately describe the rise time in all the TA kinetic traces, and so we fixed it to this value for all fits. The term in the parentheses of eqn. 1a is the weighted sum of the 2 stretched exponentials representing the fast and slow decay of the TA signals arising predominantly from the Coulombically-free (subscripts of 1) and trapped (subscripts of 2) polarons, respectively.[4]

Nonlinear least squares regression was used to find the parameters giving the best fit to the data (**Figures S5.1-S5.2**), and all fit parameters are listed in **Tables S5.1-S5.2**. We found that the β and τ parameters were highly correlated and therefore fixed the β values of the fast and slow decay components. Transport-limited processes in *d*-dimensions typically exhibit $\beta = d/(d+2)$ dependence,[4] and so we fixed the β_1 value to ½ (2-dimensional transport) and the β_2 value to ⅓ (1-dimensional transport).

In our prior work,[4] we demonstrated the "average relaxation time", $\langle\tau\rangle$, of each stretched exponential function as a good metric describing the degree of trapping of the polarons, and each is calculated using their respective τ and β values from the fits,

$$\langle\tau\rangle = \frac{\tau}{\beta}\Gamma\left(\frac{1}{\beta}\right), \tag{2}$$

where Γ is the gamma function. Lindsey and Patterson provide extensive details on stretched exponentials and their connection to the Williams-Watts distribution,[5] which we also described in the supporting information of our previous work.[4] The calculated $\langle\tau\rangle$ values are included in **Tables S5.1-S5.2** below.

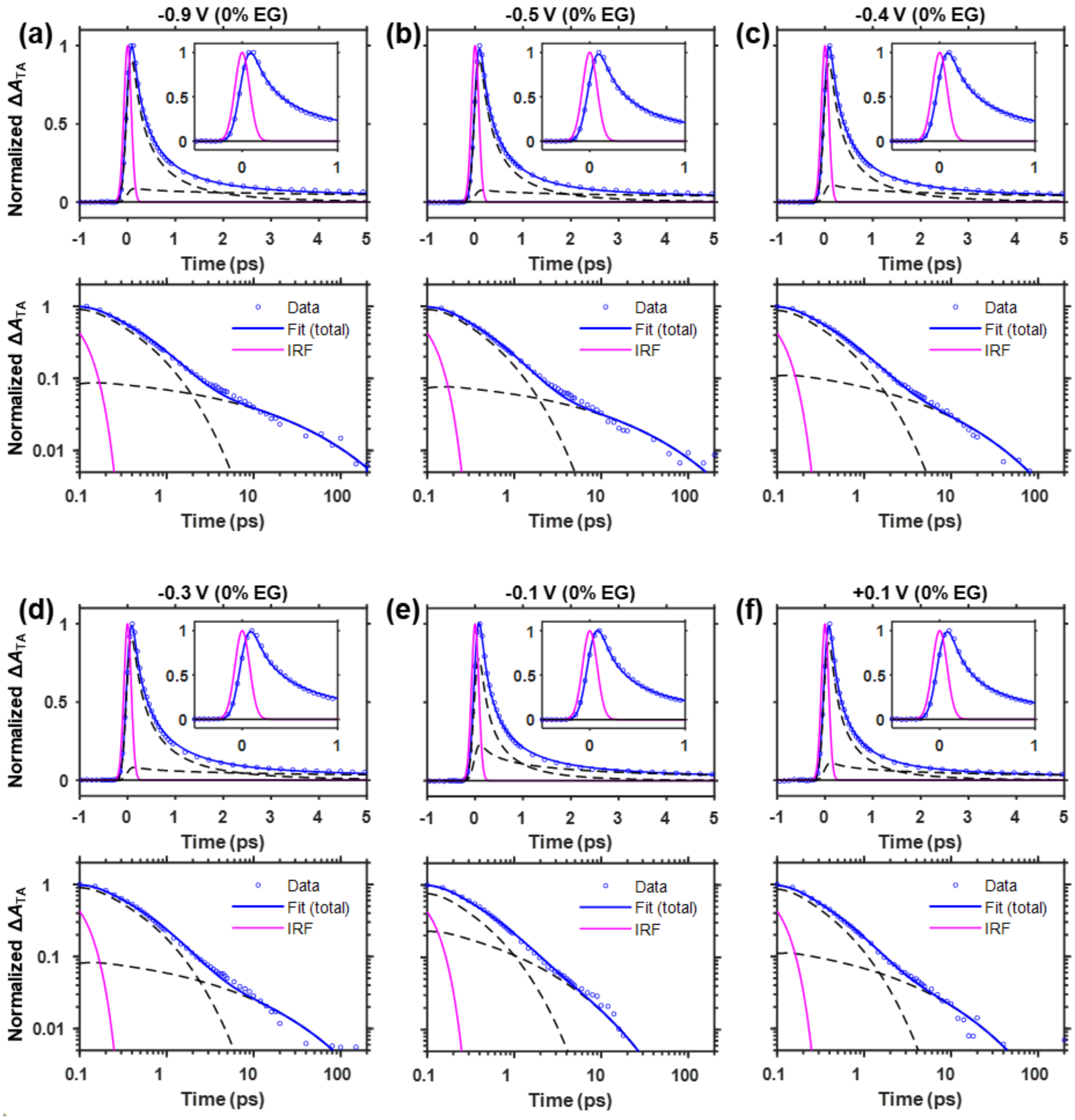

Figure S5.1. Kinetic modeling of the TA kinetics taken at the peak of the PIA band for the 0% EG PEDOT:PSS film as a function of applied voltage (vs Ag/AgCl) and presented on both linear and logarithmic axes. The data are shown as circles and the best fits are shown as solid blue curves. The dashed gray curves are the individual stretched exponential components scaled by w_1 or $(1 - w_1)$ and convoluted with the IRF (pink curves) for the fast and slow decay components, respectively.

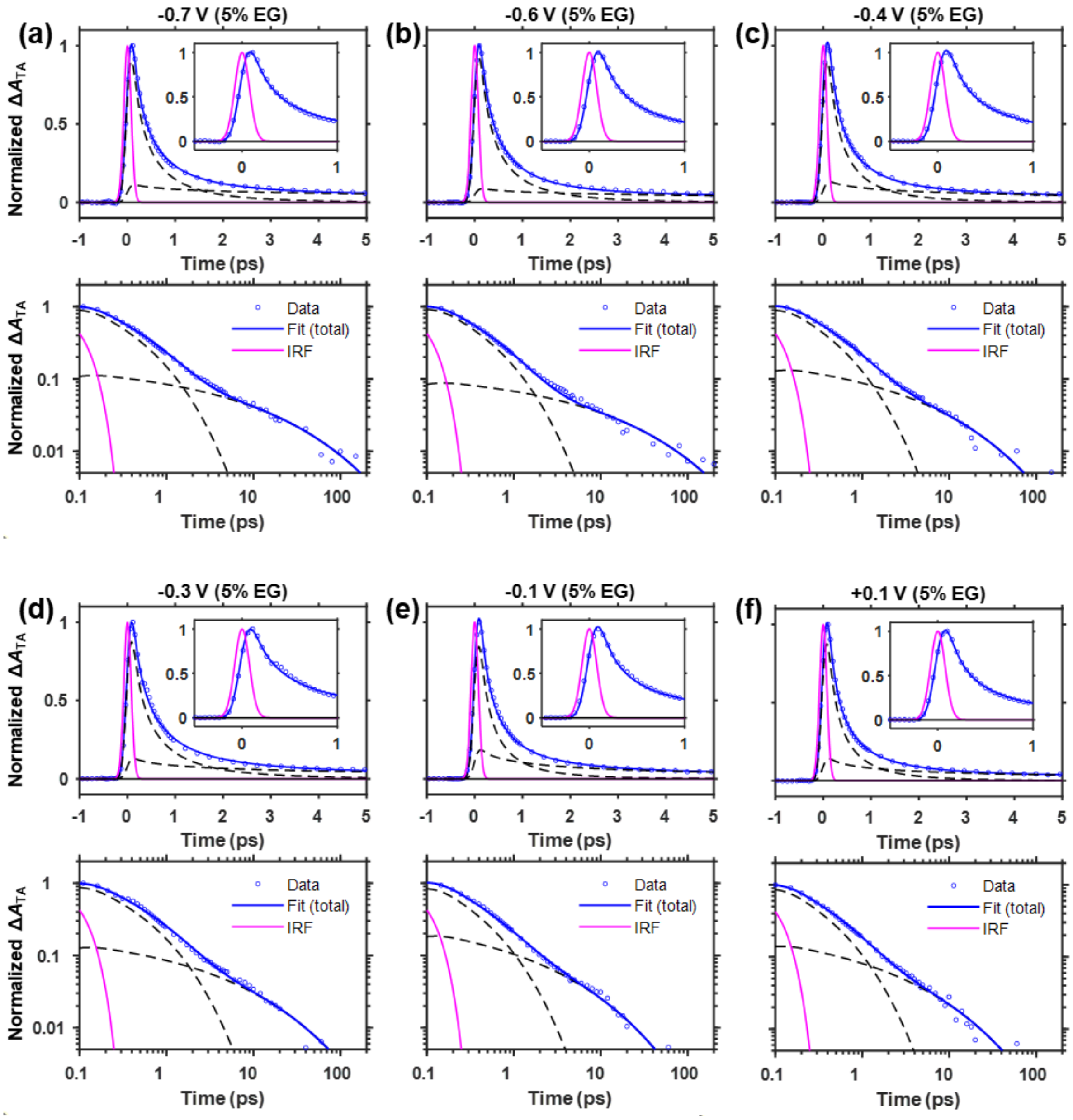

Figure S5.2. Kinetic modeling of the TA kinetics taken at the peak of the PIA band for the 5% EG PEDOT:PSS film as a function of applied voltage (vs Ag/AgCl) and presented on both linear and logarithmic axes. The data are shown as circles and the best fits are shown as solid blue curves. The dashed gray curves are the individual stretched exponential components scaled by w_1 or $(1 - w_1)$ and convoluted with the IRF (pink curves) for the fast and slow decay components, respectively.

Table S5.1. Kinetic modeling fit parameters for the 0%EG PEDOT:PSS film*

Parameter	-0.9 V	-0.5 V	-0.4 V	-0.3 V	-0.1 V	+0.1 V
N_1	2.261 ± 0.002	2.434 ± 0.003	2.307 ± 0.002	2.200 ± 0.002	2.508 ± 0.004	2.494 ± 0.003
w_1	0.9488 ± 0.0002	0.9568 ± 0.0002	0.9256 ± 0.0003	0.9440 ± 0.0003	0.804 ± 0.002	0.9234 ± 0.0007
β_1	½ (fixed)	½ (fixed)	½ (fixed)	½ (fixed)	½ (fixed)	½ (fixed)
τ_1 (ps)	0.1507 ± 0.0003	0.1317 ± 0.0003	0.1412 ± 0.0002	0.1641 ± 0.0003	0.1147 ± 0.0004	0.1115 ± 0.0003
β_2	⅓ (fixed)	⅓ (fixed)	⅓ (fixed)	⅓ (fixed)	⅓ (fixed)	⅓ (fixed)
τ_2 (ps)	7.39 ± 0.04	5.67 ± 0.04	1.80 ± 0.01	2.49 ± 0.03	0.28 ± 0.03	0.92 ± 0.01
$\langle\tau_1\rangle$ (ps)	0.301 ± 0.001	0.263 ± 0.001	0.282 ± 0.001	0.328 ± 0.001	0.229 ± 0.001	0.247 ± 0.001
$\langle\tau_2\rangle$ (ps)	44.4 ± 0.3	34.1 ± 0.3	10.80 ± 0.06	15.0 ± 0.2	1.67 ± 0.02	6.63 ± 0.08

**Uncertainties were calculated from the 95% confidence intervals for the fit parameters. The average relaxation times were calculated as described above.*

Table S5.2. Kinetic modeling fit parameters for the 5%EG PEDOT:PSS film*

Parameter	-0.7 V	-0.6 V	-0.4 V	-0.3 V	-0.1 V	+0.1 V
N_1	2.353 ± 0.002	2.505 ± 0.003	2.650 ± 0.004	2.217 ± 0.002	2.747 ± 0.004	2.616 ± 0.002
w_1	0.9335 ± 0.0002	0.9511 ± 0.0002	0.9188 ± 0.0005	0.9068 ± 0.0005	0.8719 ± 0.0008	0.9032 ± 0.0006
β_1	½ (fixed)	½ (fixed)	½ (fixed)	½ (fixed)	½ (fixed)	½ (fixed)
τ_1 (ps)	0.1364 ± 0.0003	0.1288 ± 0.0003	0.1139 ± 0.0003	0.1611 ± 0.0004	0.1026 ± 0.0003	0.1030 ± 0.0002
β_2	⅓ (fixed)	⅓ (fixed)	⅓ (fixed)	⅓ (fixed)	⅓ (fixed)	⅓ (fixed)
τ_2 (ps)	4.16 ± 0.02	4.72 ± 0.04	1.35 ± 0.01	1.42 ± 0.01	0.548 ± 0.005	0.678 ± 0.005
$\langle\tau_1\rangle$ (ps)	0.273 ± 0.001	0.258 ± 0.001	0.228 ± 0.001	0.322 ± 0.001	0.205 ± 0.001	0.206 ± 0.001
$\langle\tau_2\rangle$ (ps)	25.0 ± 0.1	28.3 ± 0.2	8.16 ± 0.07	8.51 ± 0.07	3.29 ± 0.03	4.07 ± 0.03

**Uncertainties were calculated from the 95% confidence intervals for the fit parameters. The average relaxation times were calculated as described above.*

Section S6. References

1 AR. Umar, A.R.; Dorris, A.L.; Kotadiya, N.B.; Giebink, N.C.; Collier, G.S.; Grieco, C. Probing Polaron Environment in a Doped Polymer via the Photoinduced Stark Effect. *J. Phys. Chem. C* 2023, **127**, 9498–9508.

2 Dorris, A.L.; Umar, AR.; Grieco, C. Ultrabroadband Near-Infrared Transient Absorption Spectrometer with Simultaneous 900–2350 nm Detection. *Appl. Spectrosc.* 2024, **78**, 1043-1050.

3 Phillips, J.C. Stretched exponential relaxation in molecular and electronic glasses. *Rep. Prog. Phys*. 1996, **59**, 1133–1207.

4 Umar, AR.; Dorris, A.L.; Grieco, C. Photoexcited Polaron Relaxation as a Structurally Sensitive Reporter of Charge Trapping in a Conducting Polymer. *Adv. Funct. Mater*. 2024, **34**, 2407181.

5 Lindsey, C.P. and Patterson, G.D. Detailed comparison of the Williams–Watts and Cole–Davidson functions. *J. Chem. Phys.* 1980, **73**, 3348-3357.
